# Social justice with harm reduction approaches for mothers who use opioids: an international comparative ethnographic study with community engaged methods

**DOI:** 10.1186/s12954-025-01309-6

**Published:** 2025-12-24

**Authors:** Miriam W. Boeri, Aukje Lamonica, Anne Whittaker, Marisa Bruce

**Affiliations:** 1 North Jersey Community Research Initiative (NJCRI), 393 Central Avenue, Newark, NJ 07103 USA; 2 Public Health, Southern Connecticut State University, New Haven, CT 06515 USA; 3 Nursing and Clinical Academic in Applied Substance Use Research, Faculty of Health Sciences and Sport, University of Stirling, Pathfoot Building, Stirling, FK9 4LA Scotland, UK; 4 University of Edinburgh, Edinburgh, Scotland, UK

**Keywords:** Mothers who use drugs, Opioids, Child protection, Harm reduction, Community engagement

## Abstract

**Background:**

Mothers and pregnant women who use opioids are particularly vulnerable. Mothers often fear surveillance, stigma, and loss of custody of their children when seeking treatment. Although opioid agonist therapy (OAT) has been shown to be effective, access varies regionally, and not all mothers can cease using opioids. This study compares outcomes of mothers who use opioids in the UK with universal healthcare and OAT access, and mothers in the U.S. with restricted healthcare and OAT access, focusing on their interactions with services.

**Methods:**

This is a secondary data analysis of two studies on mothers who use opioids. Data were collected from nine mothers in Scotland (UK) and 20 mothers in New Jersey (US) through ethnographic, longitudinal studies spanning one year. The UK study used a “Learning Alliance” engagement approach, a patient/public engagement model that involves stakeholders in developing objectives and the dissemination of findings. The US study engaged “community-based consultants,” who are paid individuals with lived experience from the study field communities to assist researchers in recruitment and ethnographic fieldwork. Ethical approval was received from review boards. Data were anonymized before analysis, and people with lived experience provided feedback on findings. Grounded theory methods were used for analysis.

**Results:**

Findings reveal both convergent and divergent experiences. Mothers in Scotland had more access to healthcare and social housing but faced increased surveillance, while New Jersey mothers often experienced housing insecurity and difficulty obtaining healthcare. Shared challenges included trust issues, stigmatization, inconsistent practitioner engagement, responsibilization, and unclear expectations from child protection services. While Scottish mothers had better access to OAT, both groups faced child custody loss due to unregulated drug use. Mothers in both studies were struggling to meet reunification requirements of abstinence (with or without OAT) within the required time frame.

**Conclusions:**

Differing governance structures create persistent challenges across national boundaries. While health practitioners generally support harm reduction strategies, it does not go beyond OAT for mothers. Our findings indicate the need for radical harm reduction approaches with social justice for mothers who use drugs, including safer parental drug use strategies.

## Background

Mothers and pregnant women who use opioids are a particularly vulnerable group since they fear intrusion of child protection services that can lead to losing child custody [1, 2]. Substance use during pregnancy and postpartum is one of the key indicators of child protective services becoming involved over concerns of harm to the fetus, adverse neonatal outcomes, and child neglect [3, 4]. However, co-occurring issues such as poverty, unstable housing, mental health concerns, and social isolation diffuse the link between substance use and adverse child and parenting outcomes, making it difficult to draw a causal connection [5].

Greater surveillance and stigmatization by social service and healthcare staff present barriers to seeking necessary treatment [6, 7]. Surveillance of mothers who use drugs is often sanctioned under the guise of “child protection” and involves scrutiny and monitoring of the mothers’ behaviour and “lifestyle” by social services, healthcare providers, the police, and other agencies. Surveillance practices usually focus on identifying “risks” and “harms” to children that are deemed to be attributable to the conduct of mothers who use drugs. For mothers this may mean, for example, complying with drug testing and program attendance requirements, unannounced home visits, supervised child contact, video observation in residential settings, and other intrusive and coercive methods of policing mothers [8]. There remains a gap in understanding how increased surveillance affects desired outcomes for mothers [9, 10].

This is a secondary data analysis of two studies with a focus on the mothers’ interactions with governing agencies and outcomes of child protection agency involvement. A comparison of mothers under child welfare governance in two countries with different policies and practices can shed light on the impact of increased surveillance. The two studies examined here used longitudinal ethnographic methods. One was conducted in Scotland, UK, and the other in New Jersey, US. Both studies involved community stakeholders and people with lived and living experience in study design, data collection, and analysis.

The aim of this transatlantic comparative analysis was to examine the lived realities of mothers navigating surveillance by child protection authorities, substance treatment programs, or other health and social care services, and the effects of increased governance on mothers as it related to family preservation.

### Original study settings in Scotland and New Jersey

The *Governing Parental Opioid Use: A Relational Ethnography,* hereafter called the *Relations Study*, was a longitudinal study conducted in England and Scotland. Data were collected between 2021 and 2022. Because of the differences in policy and practices between England and Scotland, only the data from Scotland are used in this US-UK comparative study.

The *Suburban Opioid Study-Providing for Opioid-using Mothers and Pregnant Women who need Treatment* (SOS-PrOMPT), hereafter called the *Mothers Study,* was a longitudinal study conducted in Connecticut and New Jersey. Data were collected between 2021 and 2022. Because of the differences in policy and practices between Connecticut and New Jersey, only the data from New Jersey are used in this US-UK comparative study.

While all mothers in both studies were considered poor, according to the standards of the respective countries, the socio-economic situation of the mothers in Scotland was better in terms of housing and social welfare. All Scottish mothers were housed, received social welfare benefit payments, and had access to free healthcare and support services. Most of the New Jersey mothers were housing insecure, on waiting lists for housing, and with few benefits. The social welfare and housing differences reflect the distinctions between the field sites in policy and practice. However, the availability of unregulated drugs and rates of overdose were similar in Scotland and New Jersey at the time of the study.

### Opioid use comparison

Parental substance use refers to the use of different kinds of unregulated drugs, including alcohol, and often involves polydrug use. Here we use the term unregulated drugs to distinguish drugs supplied through unregulated sources from drugs supplied through a medical provider [11]. The studies here focused on parental use of unregulated opioids, including heroin, fentanyl, opioid pain prescriptions, and opioid substitution medications. The opioid epidemic affected the US more than any other country, but many countries have been impacted by waves of opioid use [12, 13]. The US has the highest overdose death rates in the world, and Scotland has the highest drug-related death rates in Europe [14].

Over the past three decades, parental opioid use is increasingly viewed as a growing problem in both the US and UK. Despite a lack of systematic reporting of harms caused by parental drug use, children living with parents who use unregulated opioids are considered at risk of abuse and neglect [15]. Problem opioid use, identified medically as opioid use disorder (OUD), is a chronic condition, and relapse rates have been shown to be as high as 91%; yet studies indicate that child service agencies are often pressured to find permanent placement for children within a timeframe insufficient for parents' recovery [16].

In the US, based on data from 2017, an estimated 1.4 million children were living with a parent with OUD, 240,000 children had a parent die by overdose, and 325,000 were living in foster or kinship care due to a parent’s OUD [17]. In New Jersey, 68,500 children were impacted by parental OUD and 47,000 were living with a parent with OUD [17].

In the UK, based on data from 1996 to 2000, an estimated 200,000 to 300,000 children in England and Wales and 41,000–59,000 children in Scotland had parents with problem drug use [18]. In Scotland, data on parental opioid use in child protection cases are not recorded and reported routinely, so there are not good estimates of the size of the problem.

Opioid agonist therapy (OAT) is effective in reducing the use of unregulated opioids [19, 20]. In Scotland, OAT was free to obtain via drug treatment services or general practitioners (GP) and dispensed via community pharmacies. In New Jersey, access to OAT was difficult for many of the mothers due to bureaucratic requirements and lack of clinics approved to dispense OAT.

### Policy and practice comparison

The key difference in social welfare policies between the UK and the US is access to healthcare and social benefits. The UK provides free universal healthcare to all UK citizens through the government operated and tax funded National Health Service (NHS). The US does not have universal healthcare but a mix of private health insurance and public programs, such as Medicare and/or Medicaid for older people, people with disabilities, and those with very low income. The US is the only wealthy nation which does not provide universal healthcare and where a large proportion of people do not have access to health insurance [21].

While social welfare policies in the UK and US appear similar on paper, in practice the UK offers a wider range of social benefits to the unemployed and those with low income, people with disabilities, parents with children, and the elderly. These benefits can include economic assistance, housing support, and employment assistance. The US provides minimal benefits and only temporary financial aid or food assistance for very low-income individuals and families. Public (social) housing is difficult to access in the US and includes a mix of subsidized public housing and vouchers for private housing, often found in economically disadvantaged neighbourhoods. Eligibility for these programs is restrictive, and access varies across states. Social welfare or “safety nets” are increasingly inadequate to meet the need [22].

Both countries have policies that require oversight of mothers reported to be using substances [23–25]. Pregnant mothers who use substances or opioid prescription drugs are placed under governance of Child Protection Services (CPS) in both the UK and US. CPS might also be involved in incidences where mothers of children come under the lens of other government agencies (e.g., domestic abuse reports via police). Failure to comply with CPS regulations can result in losing custody and legal removal of children [26]. Legislation and guidelines in both Scotland and New Jersey emphasize family preservation; yet recurrent care proceedings in which parents lose one or more children to foster care, kinship care, or adoption is increasing in both the UK and the US [27, 28].

### Theoretical frameworks

The theoretical framework for the *Relations Study* drew on a range of post-structural social science theories. One of the guiding analytic approaches was “What is the Problem Represented to Be?” (WPR), which focuses on how so-called “problems” are conceptualized, problematized, and responded to in practice [29]. Bacchi proposes that interrogating the political and ethical implications of problem representations provides a basis for developing interventions that produce *‘‘as little domination as possible’’* (p.142). Starting from the premise that policy is “not a reaction to ‘problems’ […] rather policies produce or constitute ‘problems’ as a particular kind of problem” (p.131), the *Relations Study* asks how parental substance use is represented in policy and enacted in practice. One aim of the *Relations Study* was to advance theoretical understandings of how parents who use drugs are governed, accounting for the wider social ecology within which families are embedded [30].

The *Mothers Study* used a socio-ecological framework developed for the opioid crisis, which focused on complex interrelationships between individual, interpersonal, community, and societal factors [31]. One aim of the *Mothers Study* was to determine the impact of governing agencies on mothers who used opioids and their families. The theoretical frameworks of the two studies were similar in their focus on policies and practices governing parental drug use within the lived experience of mothers' social reality.

## Methods

This secondary data analysis compares data from the *Relations* and *Mothers* studies. Ethical approval for the *Relations Study* was obtained from the NHS Research Ethics Committee [Ref: 21/NS/0029] and included consent to publish and archive research data for future use. Institutional Review Boards at Southern Connecticut State University [#354] and North Jersey Community Research Initiative [#00001870] approved the *Mothers Study*. Data collection and anonymization of the data in both studies were completed before the secondary data analysis. The analysis for this paper focused on how policies functioned in practice and how practices were experienced by mothers.

### Comparison of methodological approaches

The *Relations Study*, drawing on “relational ethnography” [32], collected data on relational processes and mechanisms through observations of parents interacting with health and social care services, and interviews with mothers, fathers, and service providers. Parent participants were recruited from health and social care services via practitioner and self-referral using poster adverts, flyers, and through snowballing method. Participants provided consent for collecting confidential data. Parent participants received gift vouchers for every interview and/or month they remained in the study. They were also offered smart phones and mobile data if required.

In the *Mothers Study,* participants were recruited through street encounters, flyers, and community consultants. The ethnographic methods required researchers to become familiar in settings where they could meet and talk with mothers who use opioids, also called the “field,” including in and around treatment centers, methadone clinics, shelters, and harm reduction centers. Participants read and signed a consent form explaining the study and confidentiality of the data. They received monetary compensation for participation in up to three interviews, and they were provided with a smartphone and a monthly service plan to stay in contact with the researchers over one year.

The secondary data analysis used a Grounded Theory Method approach for analysis of the data [33]. Grounded theory is inductive, allowing the findings to emerge from the data rather than be forced into an established theory. Analysis involved coding the data to identify common themes in the lived experiences of mothers interacting with providers. We started with open coding whereby themes were identified in line-by-line reading of all transcripts and fieldnotes. Each case was read by at least two coders (double-coded). Emerging themes were discussed, defined, and merged into a unified coding scheme after four cases (two from each study site) were double-coded. The coding scheme was discussed by the authors and organized for a cohesive presentation of findings, integrating the theoretical frameworks of the studies. Case studies provided rich, detailed, context-specific narratives for more in-depth understanding of mothers’ lives [34].

### Community engagement

The *Relations Study* collaborated with key stakeholders using *Learning Alliance* methodology, a community-engagement model [35]. This involved engaging stakeholders, such as the service users (parents), family members (young people and kinship carers), front-line practitioners, service managers, and policymakers, over the lifetime of the project to exchange ideas and experiences, discuss and review study findings, and co-produce study outputs.

The *Mothers Study* included paid community consultants, who are people from the community with lived and living experiences who assisted with recruitment and accompanied researchers to areas where they might meet people interested in the study. Establishing rapport through community-based consultants has been shown to increase validity and engender trust [36].

Practitioners and people with lived and living experiences provided input and insights throughout the secondary data analysis. Initial findings were shared with community stakeholders to query their views on our interpretations. Specifically, mothers with lived experience of child custody issues due to substance use were asked to provide their insider knowledge on suggestions for enhanced harm reduction strategies.

### Positionality and reflexivity

The authors are all middle-class, white, cisgender women with different cultural and educational backgrounds. All worked in academic institutions as professors and researchers or in clinical positions. All have experience working with different populations of people who use opioids. The lead author had family members who were involved in opioid use over a lifetime. The authors approached their work with an awareness of the social, cultural, and economic privileges that shaped their perspectives. The analytic process involved the four authors reflecting on their positionality to discuss and refine the codes, themes, and interpretation of the data. They continually reached out to mothers with lived experiences with opioid use and child protection services to gain greater insights on their interpretation of the data. Some of these “community consultants” were people they knew from previous studies. Some who impacted their process of reflexivity were mothers they met conducting everyday ethnography, which is a method of making sense of social life by closely observing and interacting with others in daily experiences.

## Results

The comparative analysis included nine mothers in Scotland and 20 mothers in New Jersey. All nine mothers in the Scotland study were white. Among the 20 mothers in the New Jersey sample, four identified as African American/Black, four as Latina/Hispanic, one as Pacific Islander, and the rest as white. The race and ethnic demographics reflect the populations of the Scotland and New Jersey field sites. Mothers in Scotland were in their late 20 s to mid-40 s and mothers of between one to five children. Four mothers were not living with at least one or more of their (non-adult) children during the time of the study. Mothers in New Jersey were in their early 20 s to late 40 s and mothers of between one to five children. Thirteen mothers were not living with at least one or more of their (non-adult) children during the time of the study. The majority of the mothers in both studies lived in areas of social deprivation, defined by respective governments as areas where people have low income and few resources or opportunities.

Our qualitative analysis revealed more similarities than differences in the mothers’ experiences with governing agencies. The differences found were related to policy and practice disparities between Scotland and New Jersey; however, interactions with governing agencies and providers were largely similar among all mothers. For the sake of parsimony, we provide only a few representative quotes from the fieldnotes and interviews to illustrate a theme. For comparison purposes, quotes are identified as a Scotland fieldnote or interview, or a New Jersey interview; direct quotes from mothers are in quotation marks when part of a fieldnote.

### Differences between Scotland and New Jersey mothers lived reality

The *key differences* between the Scottish and New Jersey mothers’ situations lay in access to services and policy practices. Scottish mothers had significantly greater access to comprehensive social and healthcare services compared to those in New Jersey. However, the enhanced access to services in Scotland was accompanied by more intensive surveillance by service providers than what mothers experienced in New Jersey.

### Housing provision

Differences in access to housing was evident by the way mothers in the two countries talked about their housing situations. In Scotland, all mothers were in social housing or obtained social housing during the study, but some desired different neighbourhoods. The ethnographer described the housing situation of one mother at the start of the study:

*[Mother’s private] landlord has asked her to leave, and she believes this is because rents are increasing and they can make more money after evicting her.... She has been bidding consistently on [social] houses in a couple of other areas closer to her mother—places that have more resources, activities and space for walks. [Mother] believes that the threat of eviction has increased her priority [for social housing] and is hopeful of hearing about a new house as soon as this week. […] She explained that social work and the housing department can pay for transport (taxis/a van) to support the move (Scotland fieldnote)*

Within three months, the mother received social housing in the neighbourhood she requested. For some mothers, the process could be stressful when trying to find housing in desired areas, such as one Scottish mother who worried about the bidding process:

*[Mother] wants to stay in the community: She talked about the “proper anxiety” of having to bid for houses in areas they did not want to live—”praying that we didnae get some of the houses that we bidded on […] it was so stressful.” Her therapist wrote a letter to the housing department, who then allowed them to narrow their bidding to only a few areas, (Scotland fieldnote)*

Two months later, this mother sent a text to the ethnographer with good news: *“I just wanted to inform you that we got offered a house yesterday! Whoop whoop whoop!”* Other mothers in Scotland had similar experiences showing that social housing staff in Scotland were relatively responsive to the mothers’ requests.

In contrast, most of the mothers in New Jersey (90%) were unhoused or insecurely housed at some point during the study, with or without children in their care. Public (social) housing was scarce. Mothers provided with housing vouchers for rent were required to find their own private housing. They were faced with a housing shortage, landlords who did not take vouchers, and long waiting lists for any available subsidy housing. One New Jersey mother who needed to find housing to regain custody of her two-year-old child explained her situation:

*Section 8 housing––forget about it. The waitlist is closed and it’s probably not gonna open anytime soon. TRA…Temporary Rental Assistance Program… It’s not closed; however, because I have used my emergency assistance twice within a 12-month calendar year, I’m not eligible to get any emergency assistance [...] So besides all that, there are subsidy housing buildings; like apartments that they will just [adjust your] rent based on your income. But my income is not enough at this point. I’ll need to get cash assistance from welfare. […] I’m most likely gonna have to get kicked outta the shelter and then wait for these places to respond about my applications. And there’s no guarantee that I’m gonna get approved for those units. There’s no guarantee that you’re gonna get what you’re tryin’ to get. You might lose everything. Plus, they have all these application fees and I’m like…if this is income-based livin’ why’s the fee $100? I don’t have $100 just to put an application in and I might not ever even hear back.” (New Jersey interview)*

Other New Jersey mothers experienced similar lack of help from social housing services, who rarely interacted with them. Unhoused mothers in New Jersey were required to go to various social service offices to apply for any services available to them. This usually required getting an appointment. One unhoused mother explained the barriers of trying to obtain social housing:

*“I don't have an I.D. Also, I don't have a phone, which they’re like ‘we're going to call you back to the appointment.’ There's no way to call me back... Then I have to get there every day. I don't have transportation … Everybody wants you to go get help. Nobody wants to help you when it comes down to it.” (New Jersey interview)*

As illustrated above, mothers in Scotland and New Jersey described distinct differences in such a basic social need as housing, but the easier access to services came at a price.

### Service providers

Whereas the New Jersey mothers expressed a difficult time getting help from social services and rarely had contact with social or CPS workers, mothers in Scotland had so much input from so many service providers they felt overwhelmed.

Scottish mothers under CPS were provided multiple “support services” integrated through a Child Protection Planning Meeting (CPPM) group. These included health providers, mental health and treatment practitioners, and social workers specialized for family, parent, or child. The CPPM met to discuss the child’s care plan and make decisions on starting a permanence removal process. Mothers often expressed being surprised at the negative reports they read or heard from support workers at these meetings. For example, one mother described a meeting discussion of a comment from a pre-school teachers' report:

*“I was quite annoyed about them saying that [pre-school age child] was unkempt cause it’s like but you tell us to put old clothes on them [for pre-school]… cause the woman’s like ‘well maybe about ironing or things?’ Why am I going to iron jogging bottoms and sweatshirts? [...] and I felt like, what––just cause I have social work involved yous are picking at little things? And that’s what it felt like, that they were talking behind my back and picking at little things?” (Scotland fieldnote)*

A Scottish mother of a young child described what it was like under surveillance after she brough her newborn home:

*“I had everybody. Oh, it was ridiculous, and I had the social worker, I had the [pregnancy/drug support service]. I had the midwife. I had health visitors. Yeah, the drug worker, she came in with [pregnancy/drug support service] but it was all different appointments. I had so many appointments, it was ridiculous, and my head was spinning wi’ it all, and I think [Addiction Nurse] realised, I told [her] ‘it’s getting too much all these people every day poking their nose in and checking in.’ […] One day I ignored my door 'cause I just couldnae be bothered with them, but then I heard the shout through the door: ‘If you don't answer the door we might have to phone the police, 'cause she’s on the child protection.’” (Scotland interview)*

As shown in this quote, having so many support workers also meant more surveillance of the mothers from providers with influence over child custody decisions.

The enhanced Scottish support services was a double-edged sword, with remarkable access to needed services, such as housing, childcare, healthcare, treatment, and transportation on one side, and intensive surveillance by providers on the other. In comparison, access to support along with increased scrutiny was largely absent for New Jersey mothers.

### Similarities among Scotland and New Jersey mothers lived reality

*Similarities* between the two samples of mothers emerged from their lived experiences in interactions with practitioners and providers. These were grouped into themes described as issues of trust, pervasive stigmatization within care systems, inconsistent or lack of engagement from professionals, the burden of responsibilizing the mothers, and contradictory messages.

### Trust

Mothers from both studies expressed serious trust issues with providers, often learned through past experience. A Scottish mother described how she lost trust in social workers due to a breach of confidentiality where she lived previously:

*“I told them ‘I do not want my brother or my sister-in-law to know about my past’ and then the social worker actually went in front of me, this is [social worker in previous location], she went in front of my sister-in-law, cause I went ‘I've told her about I'm getting support’ and she went ‘oh so you've told her about the drugs?’ And I was like ‘no I didn’t, like I just said I told her I'm getting support’. Like, she told things in meetings and things without my permission […] So that’s why it’s very hard to now have trust in the social work here.” (Scotland interview)*

Likewise, a New Jersey mother revealed how her trust in her therapist was lost:

*“She [therapist] was talking to a—I was in a shelter in [County] for a little bit––and she was talking to the shelter director [about my mental health] without my permission. And it just broke my trust with her… I do need to go back to therapy. I was in therapy for a while [with her] but she broke my trust.” (New Jersey interview)*

While some incidents with trust occurred in the past, they impacted mothers in the present. Mistrust of service providers can engender feelings of judgement and stigma, provoking mothers to avoid needed services.

### Stigma

Despite ongoing efforts to reduce stigma toward people who use drugs, the data show that it remains pervasive among social and healthcare providers. Mothers who use drugs are among the most heavily stigmatized, and they go to great lengths to avoid feeling judged. Many of the Scottish mothers expressed the stigma they felt when picking up their OAT at the pharmacies:

*“Even at the chemist [pharmacy] you get a stigma about the drugs. I feel like, 'cause you get treated completely different. Yeah, like people get served quicker when they're not on like controlled drugs ... [The pharmacy] opens at half-eight but we are not allowed to go up till half-nine, and like it closes at six but we're not allowed to go, we've got to be there before five.” (Scotland fieldnote)*

In New Jersey, mothers expressed feeling so stigmatized by providers in the past that the shame prompted them to hide current or past unregulated drug use. One mother described how she felt when revealing her drug use to health providers:

*“My teeth are terrible. I don’t know if it’s from drugs. But I tell them [doctors] that I use, and that’s probably why my teeth are that way. I get some looks. So, that makes me feel really small.” (New Jersey interview)*

### Lack of engagement by providers

Mothers in both studies reported difficulty reaching providers or social workers when needed. Scottish mothers were kinder in their judgement of lack of engagement, often finding excuses for their providers––nevertheless feeling “let down.” As a Scottish mother illustrated:

*“My doctor says I'm one of these people that have somehow, keep falling through the cracks…. Yeah, because people are letting me down, and he [doctor] says there’s a lot of people keep letting me down.” (Scotland interview)*

New Jersey mothers joined the chorus decrying providers who never called back, did not respond to multiple applications, or sent them elsewhere. As one mother lamented:

*“She (case worker) helps here and there, but…she helps as far as tellin’ us…where to go to get services, where to go to get in a program. Anything else as far as housing and all, she’s like, ‘Call social services.’ That’s it. That’s as far as she go.” (New Jersey interview)*

### Responsibilization of mothers

This term is used to describe a process in which mothers are held responsible for managing their own risks, health, and well-being, and the health and wellbeing of their children, shifting accountability away from institutions, governments, or systemic factors that play a key role in shaping family life and the welfare of families [37]. The process of responsibilization leads to interventions that focus on individual behaviour change and not interventions that address wider social and systemic problems. Mothers from both Scotland and New Jersey were unaware that they were experiencing this phenomenon, and they often accepted that they were responsible for decisions made by providers. Responsibilization is shown in what mothers were told by CPS/CPPM:

*“The ball is in your court.” (told to mother by Scotland social workers after CPPM decision to start a permanency process.)*

The above idiom implies that the mother bears sole responsibility for subsequent course of action, effectively absolving social and healthcare agencies of accountability for further intervention toward family preservation. In New Jersey, the mother’s accountability is implied from the start:

*“We don’t help with housing at all. If you don’t have somewhere to live, you’re not gonna be able to get [your son] back. You gotta get yourself somewhere to live. You gotta get yourself a job, you gotta get yourself sober, and you gotta have all of that before we give him back to you” (told to mother by New Jersey CPS case worker.)*

### Contradictory practices

Despite policy guidelines in both countries emphasizing collaboration with families and clear communication about reunification processes, many mothers reported confusion or received contradictory directives. For example, a Scottish mother who desperately desired contact visits with her children received opposing messages from social workers of the same agency:

*This previous social worker had been resistant to [mother] having any contact with the children - “he wouldnae let me have anything to do with [the two older children]” - but his replacement, the children’s current social worker, facilitated contact video calls, then face-to-face meetings every other month (Scotland fieldnote)*

The contradictory practices may stem from ambiguous policies. While family preservation and a path to reunification are stated goals of child protection policy, many mothers were not offered guidance from CPS on how to achieve reunification. One New Jersey mother was seeking a lawyer to be reunified with her five children. When ask if she received support from the CPS, she replied:

*“No. They were trying to do the complete opposite. They were trying to take my kids permanently away* […] *Once I get an apartment, then I’m gonna look into legal help, but I don’t wanna go the legal aid [government] help route. I wanna get a paid attorney.”*

Permanent loss of child custody decisions are required within specific time frames and, therefore, do not leave time for relapse, a common process on the path to recovery. When challenged about “positive drug tests,” mothers struggled to achieve abstinence within the limited time frames required. Two case studies compare the challenges mothers faced trying to be reunified with their children once they were under CPS.

### Case studies of child protection experiences in Scotland and New Jersey

To better understand the complex processes involved and the impact of governing agents on the mothers' lives, two case studies are presented which include content-specific details that are not apparent in a thematic analysis. Case studies provide detailed insights of lives over time, offering a deeper understanding of the multi-faceted environments and entangled relationships of individual situations [34].

The following is a comparison of a mother in Scotland and a mother in New Jersey, both of whom were separated from their child by CPS. These two mothers are compared because they are the *only* mothers with a final decision made regarding return of their child within the time frame of the studies, offering invaluable real-time experiences. Other mothers involved with CPS did not have final decisions made during the study period. Their lived experiences provide more nuanced insights, showcasing the incongruities of practice and ineffectiveness of policy for family reunification and well-being. Names used here are pseudonyms.

### Donna (Scotland)

This mother in Scotland used heroin before she learned she was pregnant. At the start of the study, Donna was living in social housing with her partner, the father of her newborn baby.

*[Donna] explained that she was with a homeless GP practice receiving “drug misuse” support, and that when she found out she was pregnant, social work become involved automatically, “right away.” [Several] months into her pregnancy, they [social services] applied for a court order to take [baby] to foster care ‘until they see how hard we work to get her back, and they said that we’re doing very well.’” (fieldnote)*

With her infant given to a foster carer since birth, Donna and her partner prepared a room for their baby’s return. She describes her hopes:

*“I hope it’s gonnae [be] home visits with the baby in the home, seeing what’s safe, what’s not. Just their idea on certain things, does the baby get too hot, do we need some fireguards for the radiator? […] stuff like that…If I don’t get [baby] back...I wouldnae be here, do you know what I mean? There would be no—me and [partner], our lives wouldnae be worth living, know what I mean, without our baby.” (fieldnote)*

**Fig. 1:**
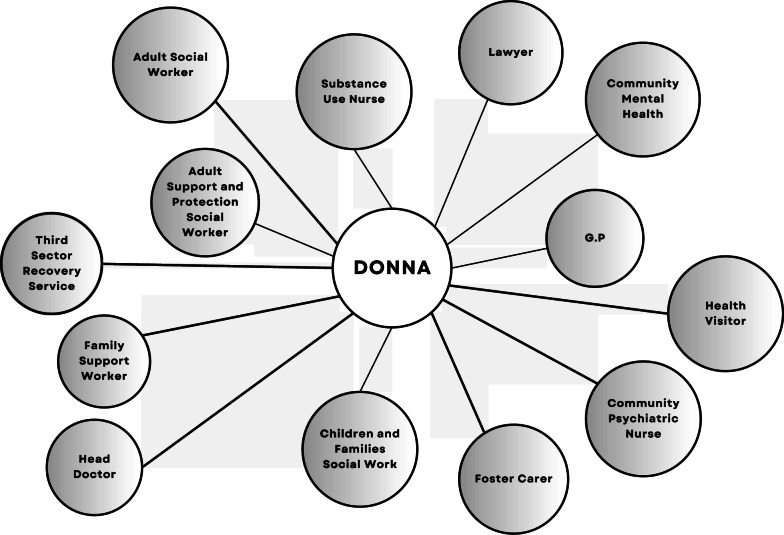
Donna’s service providers

Donna was provided a team of 13 support workers, illustrated in Fig. 1. Most of them were present at the CPPM discussions about Donna’s case.

Donna did not have a good relationship with many of the providers and practitioners governing her life. She said many came and went, and even those who were good were not consistent. Some were rude, as observed by the ethnographer:

*When Adult Protection Leader [CPPM] hangs up on Donna and ignores her phone calls Donna described this as: “worse than people would treat me when I was living in the street”, suggesting a sense of shame, stigma and frustration. (fieldnote)*

The ethnographer’s fieldnotes describe conversations and observations of Donna’s contact visits and CPPM meetings while she is trying to regain custody of her infant. She was allowed contact visits with her infant for only two hours a week in a sterile agency room with plastic chairs and no baby items. She was observed by a social worker and sometimes the foster carer was present as well. She was stressed about the visits. The baby often cried when she held him. The social worker advised her to bring a mat and toys, but when she lay the baby on the mat and tried to show him toys, he cried more. Donna suggested that the baby could have “sensory issues”, but the social worker ignored her. Concerned that her baby cries when she is with him, Donna confides:

*“it’s hard when your [baby] doesn’t want you. I dinnae know what tae dae […] I’m no’ an expert […] I feel like a fish out of water.” (fieldnote)*

Months later, the foster mother told Donna that the doctor confirmed the baby has sensory issues. Donna finally feels vindicated.

*Many of the concerns Donna had raised that had previously been dismissed—around [baby]’s sensory problems, [baby’s] crying due to teething, [baby's] discomfort in the space used for contact—had since been accepted and changes made to the contact experience. Donna says “I’ve never seen social workers who never knew what to do. […] Everything I’ve said has came, you know, has happened. When I’ve said ‘I think [baby] likes to get left on the mat’ but they were like ‘oh but you’re not engaging with [baby] properly then […] I felt like I was always getting ‘no, no, no, no, no, no, no.” (fieldnote)*

Donna also had excruciating pain, but the GP and other health providers appeared to think she was drug seeking. Months later, she was admitted to a hospital for emergency surgery. The pain and surgery caused her to miss a few contact visits.

Donna was prescribed OAT (methadone); but she still used unregulated drugs a few times with her partner. Required drug testing meant the Addiction Nurse knew of her occasional drug use. However, she told Donna she was doing better.

At the last CPPM discussion, Donna heard from the many providers she had interacted with while trying to regain custody of her infant. They were mostly negative reports. They said she missed contact visits, was visibly stressed with the baby, and was still taking drugs. The ethnographer observed:

*The Senior Children and Family Social Worker says in the meeting that Donna seems a little funny, insinuating she is on drugs. This is uncalled for and remains an issue later –Donna is on benzoa[diazephines] prescribed and these can make her seem a bit funny—she is also under tremendous pressure and has anxiety. No one at meeting mentions this. (fieldnote)*

The Addiction Nurse said that Donna did have positive drug tests, but they were much fewer than before and she was progressing. Nevertheless, the CPPM core group decided to begin the permanency process. A lack of “evidence” of abstinence from unregulated drug use appeared to be the deciding factor in initiating permanency proceedings. Donna felt significant anger feeling they had built both parents’ expectations around the return of their baby, then moved to permanency.

*“I cannae get over how they pulled the carpet beneath us like that…People don’t realise that we thought we were getting a child home […] We’re constantly walking past an empty baby room. [Permanency] is almost like a child loss […] a stillbirth or something like that.” (fieldnote)*

The parents struggled to come to terms with this decision. Donna described a lack of will to live, and her drug use intensified.

### Nancy (New Jersey)

At the start of the study, Nancy was living on the streets with her partner–– father of her child. They were previously living with their child in the home of his parents, but during the pandemic they were told to leave because the parents were old and worried about getting Covid. She explained:

*“We didn’t have nowhere to go so we just stayed in a hotel for a little bit off of the savings that we had. I kept tryin’ to get social services to give us TRA or Section-8 [public housing] or some type of housing grant, whatever. I lost my job because I started…I just started crumbling under the pressure, so I started not goin’ to work some days ‘cause I was too depressed or too anxious. I was scared and all that…I tried to go over to other shelters and most of ‘em was like, 'You can’t bring that baby in here. You have to go to a family shelter.'“*

**Fig. 2:**
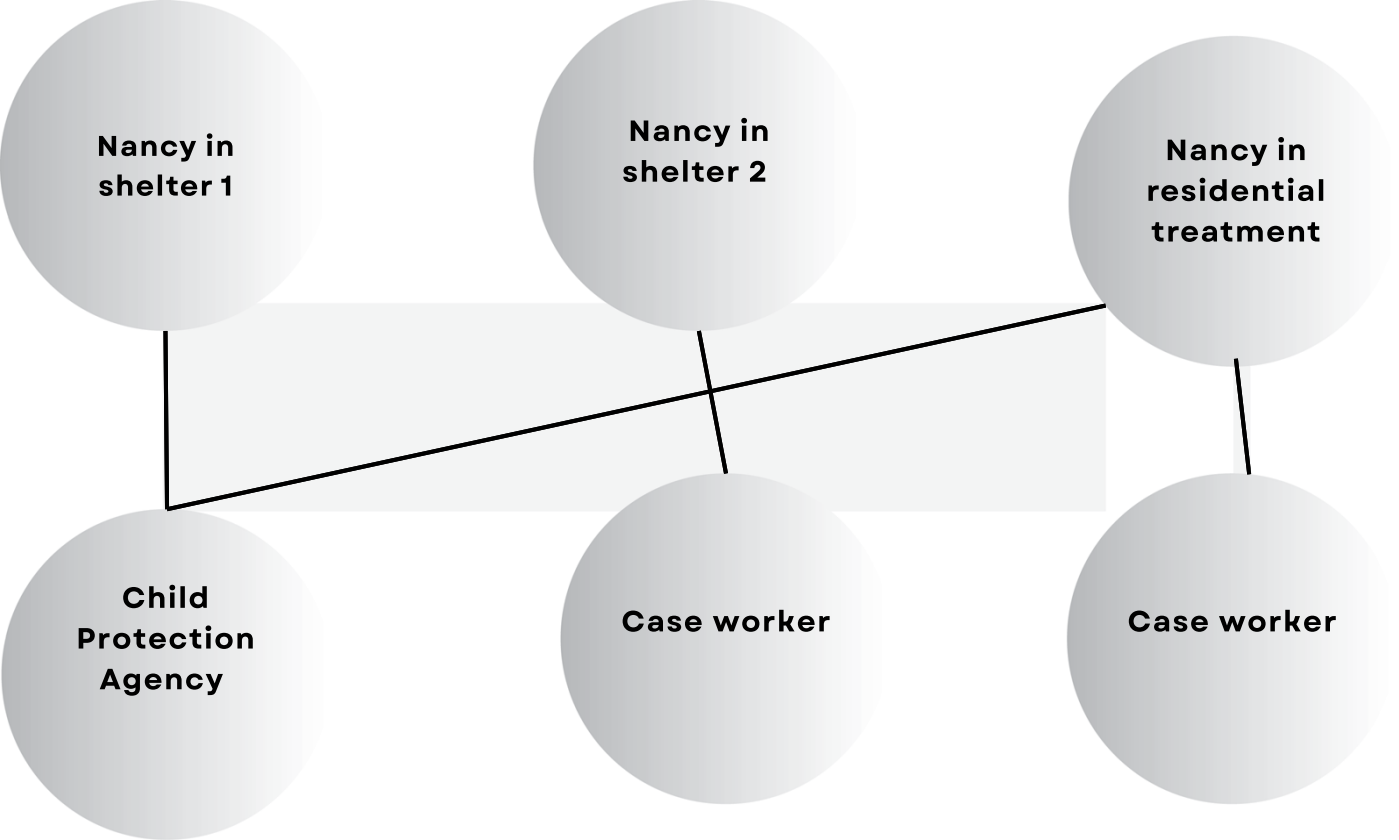
Nancy’s service providers

While Nancy was in a family shelter in the city, she overdosed in front of her toddler. CPS became involved, and Nancy’s sister received custody of her child. As illustrated in Fig. 2, Nancy has only one CPS worker, and one case worker at a time.

Nancy rarely sees the CPS worker, who told her *“Look, I’m here to help your [child]. I’m not here to help you.”* She stayed at different shelters during the time she was in the study, where she has a case worker connected to the shelter. She knows the CPS case worker talks to her shelter case worker:

*“Only thing they [shelter case workers] do as far as my CPS case, is they stay in contact with my [CPS] case worker, let her know that my urines are clean, let her know that they not havin’ no problems with me, that I’m goin’ to my groups, and stuff like that.”*

During the time Nancy is using heroin and living without her child in shelters, she spent some time in treatment programs. At one point she was seeing a psychiatrist, but she was afraid to tell him about any drug use:

*“I didn’t tell my psychiatrist I was using heroin. I definitely didn’t tell him. ‘Cause I don’t want him to tell my case worker for CPS, because she has the authorization to call him and see how I’m doing.”*

Having lived unhoused since the Covid-19 pandemic, Nancy grew more confident in her interactions with social services. When asked if social services have been very supportive, she replied:

*“Recently they have, but initially they wasn’t. Like when I first—like when Covid first hit––social services was terrible. You couldn’t—you had—I had to wait three/four months to get my food stamps... And then once I went into the program, my inpatient [treatment program], and I came out and I started feelin’ a little more confident, and I started puttin’ my foot down with them and sayin’, ‘You’re gonna do this. You’re gonna help me.’ Then they started to help.”*

Nancy must be referred by a welfare social service to get a bed in a shelter. She kept going back to the welfare office day after day because she would not take a referral to a shelter in the city again.

*“Welfare placed me in other shelters before, but they were in very bad areas and everybody around me was sellin’ drugs and everything. So every time I needed them to place me, I always told them, ‘You cannot send me here, here, here. You cannot.’ And then they will say, ‘Well, we don’t have anywhere else to send you.’ And I was like, ‘What about [town] in the suburbs.’”*

Instead of going to a shelter in the city, Nancy stayed overnight in emergency department waiting rooms when it was too cold. During the day she went to the welfare office.

When Nancy became pregnant, she was moved to the top of a waiting list for a residential treatment program for mothers with children. Here, she was able to keep her baby when she was born. She regained custody of her older child, who came to live with her at the residential treatment home.

Nancy was using OAT (buprenorphine) at the end of the study. The case worker in the treatment home helped her find temporary housing when she left the program with her two children. Her partner (father of children) is still in treatment.

Her situation is not ideal. She cannot permanently stay in her house, which is in an impoverished area of the city with high rates of drug-related crimes. She is optimistic, thinking when her partner comes out of treatment he can find work, and they can live together in a better place.

## Discussion

This comparative analysis illuminates how divergent governance structures produce distinct maternal experiences while revealing persistent challenges that transcend national boundaries. Most mothers in the study were impacted by multiple negative social and structural conditions, including health concerns, economic challenges, and social isolation. Whereas our thematic analysis identified similarities and differences in environmental conditions and social interactions with practitioners, the case studies situated individuals in the “complex social milieux” in which these conditions and interactions are “entangled rather than separate” [34, p. 2].

Based on the universal health and social services in Scotland, one would expect Scottish mothers to have better interactions with providers and less difficulty reuniting with their children than mothers in New Jersey. Moreover, mothers in Scotland had easier access to OAT than mothers in New Jersey. Yet, despite their attempts at recovery, most mothers in both Scotland and New Jersey who had children removed were unable to become reunited with children during the course of the studies. And regardless of the greater social support in Scotland, mothers in both studies experienced analogous difficulties with providers.

Like previous literature on this population, mothers in both Scotland and New Jersey felt guilty, depressed, and anxious, living with the loss or threat of losing their children [2, 38–40]. They faced stigma and seeming lack of empathy from many service providers, a finding supported in other research [6, 40, 41].

Our findings show that CPS in both Scotland and New Jersey require unregulated drug cessation within a limited time frame for reunification. Most people cannot stop drugs on demand. Recovery is a process that often involves multiple relapses and includes dimensions of health and well-being, purpose, quality of life, and connections to community, which take time to rebuild [42, 43].

Our comparative findings corroborate the results of decades of extensive research on policy and practice within substance use and recovery literature in the following areas:

1. Address ongoing stigma in attitudes of providers [19, 37, 44–48]. Mothers in both studies felt stigmatizing attitudes from healthcare and social welfare workers on an overwhelming basis.
2. Develop contextualized support services addressing maternal needs [49]. Mothers benefit from having integrated support [50–53]; however, mothers expressed a desire for help focused on their *specific* needs.
3. Increase transparency in child protection removal and reunification processes [39, 47, 54]. Mothers expressed being overwhelmed by appointments in Scotland, but like their US counterparts, they were confused and at a loss as to what they needed to do to be reunited with their children.
4. Expand social support networks in the community [53, 55, 56]. Most mothers in both studies expressed lack of friends who did not use, negligible social life, and feeling disconnected from others in the community where they lived.
5. Increase access to residential treatment homes where mothers can benefit from having holistic, integrated support while they live with their children during recovery [51, 53, 57, 58]. Although New Jersey offered residential housing for mothers and children, there was a very long waiting list with strict criteria for admittance and remaining in the program. While residential treatment for parents exists in Scotland, it appeared to be underused.

Beyond supporting existing research on improving policies and practices for parental opioid use, we sought to contribute new insights with our comparative findings. Despite policies in both countries stating that family preservation is the goal, the lived experiences with policy in practice indicates that what practitioners are doing is not working*.* Current practice is not matching policy in either country. Parental rights termination involving permanent loss of access to children is increasing while policy is emphasizing family preservation [26, 59, 60].

The case studies show that mothers experienced loss of custody or removal of their children because of their drug use and not because of evidence of child neglect or abuse. Considering that mothers who use unregulated substances but avoid detection are not subject to removal of their children, punishing only mothers who are caught, who are generally poorer and without resources, is not a socially just response. Mothers with accumulated and intersecting negative social and health conditions ought not be restricted to a one-size-fits-all model of “abstinence only” because they are under surveillance [2, 38, 61]. Further, OAT cannot be the only harm reduction strategy for mothers, when there are a multitude of other appropriate harm reduction strategies available [62].

Grounded in social justice principles that oppose the involuntary separation of children from their mothers based primarily on substance use, we sought alternatives to child removal policy and practice because of drug use only. For this, we turned to stakeholders in the community for further insights on our findings. Talking with frontline providers (e.g., social workers, homeless advocates, health providers] and mothers with lived and living experiences, we sought other approaches. Based on these discussions of our findings, here we offer feasible alternatives that go beyond simply enhancing or expanding existing models for mothers who use drugs.

### Alternatives to abstinence or OAT: a radical harm reduction approach

Removing a child from a mother who is unable to cease all unregulated substance use in a confined amount of time is not the right tool for the wellbeing of the child or mother [62, 63]. Our findings call for a more radical, transformative justice approach for mothers who use substances [64]. Querying mothers with lived experiences and applying a WPR interrogation of the “problem’” of parental substance use [29], we propose three alternative approaches for mothers: (1) adopt a “good enough” parenting perspective that does not equate parental substance use with child neglect and abuse; (2) provide moderation management; and (3) implement more radical harm reduction strategies for mothers.

### “Good enough” parenting for mothers

Not all mothers experience the same structural violence as those in our study who are under “systemic surveillance and regulation” [62, p. S192]. Some mothers have enough resources to avoid the intense scrutiny of governing agencies, and most do very well raising children while coping with drug addiction [7].

Mothers who use unregulated substances are met with a binary choice; stop unregulated use of drugs or face the risk of losing children. With this in mind, we must ask: what is the problem with mothers using unregulated drugs? What evidence is there of the “risk” to the child, which equates drug use with child neglect and abuse [10, 62]?.

A study on parents who used drugs daily found that when parents connected with peer advocacy organizations that had allowed them to develop a strong sense of identity, they were able to practice “good enough” parenting while still using drugs, contesting representations of all people who use drugs as incompetent parents [7]. The findings show that parents who use drugs employ various harm reduction strategies; however, the scarcity of positive public portrayals of safe and competent parenting by this group, compounded by punitive drug policies and the fear of intervention, ultimately hinder the creation of safe family environments.

The founder of the Harm Reduction Mother 2 Mother (M2M), a support network for mothers who use drugs, offers additional evidence that mothers who are using drugs fear engaging with doctors, treatment, and harm reduction services to avoid stigma, surveillance, and custody loss [65]. However, there are alternatives to child removal that can provide child safety as well as family preservation for mothers who use substances [62, 65].

### Moderation management

While some mothers desire to live without substance use, other mothers would prefer to learn to use substances safely or in moderation. Our own inquiries regarding acceptance of mothers using substances in a controlled manner was met with apprehension by most providers, whereas mothers with lived experiences generally agreed that moderation could be learned.

Previous studies have long provided evidence of what was called “controlled use” of drugs, including parents who raised healthy children successfully while using substances in a controlled way [7, 66]. Zinberg identified how people learned control depending on their “[mind]set” when using and the “setting” of use [67]. Studies on Moderation Management, an alternative to Alcoholics Anonymous, found that some “problem drinkers” could return to controlled drinking, while others needed abstinence-oriented interventions [68].

Controlled use of substances is not new, but it has been largely ignored. Parents who use substances but hold mainstream social roles face less scrutiny from law enforcement and CPS than resource-poor parents [66, 69]. However, current drug treatment models largely operate within a binary of abstinence or relapse, ignoring the nuanced reality of controlled use. If moderation is a learnable skill, could moderation be an acceptable harm reduction approach for mothers who use opioids?

### Harm reduction for parents

Harm reduction originated as an alternative to abstinence and ranged from safer use to managed use, with the goal “to meet individuals 'where they are at' … to minimize the harmful effects of a given behavior” [70, p. 591]. Our findings show a need for “safer drug use” harm reduction approaches for mothers who use drugs, such as the “harm reduction strategies for parents” proposed by the National Harm Reduction Coalition [71]:

- Record how much you use. This may reduce your use, even if that was not your original goal.
- Make a list of the risks and benefits of stopping and continuing your use. Think about where you’re at or who you’re with when you use.
- Switch to a safer method—which might be different for each substance. For example, taking a pill is safer than injecting heroin, but it is easier to control your dose of cannabis with smoking rather than eating edibles.
- Make a safety plan before you use. For example, arrange transportation so you don’t need to drive.
- Attend support groups like Moderation Management, SMART Recovery, Narcotics Anonymous, or Alcoholics Anonymous. Look for peer support.
- Set limits on when and where you use, like waiting until after 5 p.m. to drink or only using at home or with a trusted friend.
- Avoid using opioids, alcohol, or other depressants (downers) when you are alone or feeling vulnerable.
- Set personal limits on what you use, when you use, and how much you use. For example, don’t combine substances, or plan to have no more than three drinks over two hours.
- Make a parenting plan before any substance use—including alcohol use. Arrange for help with childcare. Know what you’d do in an emergency.
- Take good care of your body and mind. Eat healthy foods. Get enough sleep. Exercise. Drink water [71].

Moderation or safer parental drug use is currently not supported by most policymakers [72]. Policymakers and public health officials focus on the “risk of harm to children” [37], based on literature describing an association between parental use and harm advancing “the science of risk factors that is largely the science of use versus non-use” [73, p. 2]. We know parents can use unregulated substances without harm to their children [66, 74]. They can do this better and safer if unregulated substances become regulated.

For controlled or safer substance use by parents to be accepted by policymakers and practiced by child protection workers, drug policy needs to change. Legislation approaches to decriminalize or legalize drugs is the most popular way to do this currently [75].

Decriminalization is a policy-level harm reduction action that has shown to reduce stigma and structural inequities, lower rates of infectious diseases and criminal activities, and promote help-seeking [75–77]. Decriminalization would also allow mothers who controlled their drug use to maintain custody of their children, regardless of their socio-economic status. It would provide an opportunity for mothers who would like to learn moderation to acquire the skills and techniques needed to practice “safer” use.

Legalization takes this a step further to provide an opportunity for regulated use of some substances [78]. While the debate over decriminalization versus legalization is ongoing, and literature is lacking on the impact of the recent drug law reforms [77, 79, 80], years of research on legalized heroin assisted treatment (HAT) in Canada shows promising results [81, 82].

Decriminalization or legalization would reduce the environment of fear among mothers, which creates greater harm:

*Fears of child protection interventions may stop people seeking help, with the consequence that environments that could otherwise be safe for children become unsafe.”[…] Positive representations of drug use and parenting may support policy efforts to reduce the number of reports made to the child protection helpline by presenting a counter narrative to the prevailing view that drug use and parenting invariably puts children at risk [7, p. 122].*

Despite their intention to support families, current policies and practices that overlook essential life patterns of motherhood are potentially placing children at greater risks for harm [62, 73].

### Limitations

The findings here are based on women interviewed in Scotland and New Jersey, and cannot be generalized to other communities with different policies or resources. Sample size was impacted by both studies starting during the Covid period. The small sample in the Scotland study limits the range of experiences; however, the involvement of mothers with lived experiences engaging with study findings in Scotland partially addresses this limitation.

## Conclusion

In conclusion, if we value social justice, radical harm reduction for parents who use substances is a more *just* alternative to the profound *injustice* of tearing children away from their mothers. Separating parent and child even temporarily is destroying a family. Our findings challenge those who work with parents who use unregulated drugs to follow what their policies say is a primary emphasis––family preservation. Our case studies suggest that family preservation is not the focus of contemporary practices in Scotland or New Jersey. We join the call for more research using a “child-centered model” for harm reduction that advocates safe strategies for parental drug use [73].

We propose *expanding alternatives* to child removal––not merely expanding services and service providers. Some parents need more social resources and parenting support, while others might need residential parenting centers to keep families together in a safe environment while learning skills for recovery that leads to abstinence. But recovery does not always mean abstinence, and parental substance use does not always mean child neglect [7, 75, 83]. Many parents using drugs in moderation are already managing to raise their children in safe environments with healthy relationships. The harm reduction philosophy rejects a moral judgment of substance use in favor of a compassionate and functional understanding [75]. With support from mothers with lived and living experience, we are calling for a harm reduction approach that promotes safe parental drug use and good enough parenting [7, 65]. Taking into consideration the range of drug use behavior and the socio-ecological factors of mothers who are subjected to “the labyrinthine, mutating entanglement that is social and public policy” [34, p. 320], we propose alternative approaches to abstinence to stop removing children from mothers against their will. More research is needed on which alternatives work for which situations, and how to implement these options in policy and practice.

## Data Availability

The Relations Study Data, 2021–2022. [data collection]. Is available on request at UK Data Service. SN: 857,133, 10.5255/UKDA-SN-857133]. The SOS-PrOMPT data is available on request from Aukje Lamonica.

## Abbreviations

CAPTA: Child Abuse Prevention and Treatment Act
CPPM: Child Protection Planning Meeting
CPS: Child Protection Services
HAT: Heroin assisted therapy
M2M: Mother 2 Mother network
OAT: Opioid agonist therapy
TRA: Temporary Rental Assistance
UK: United Kingdom
US: United States (of America)
WPR: “What is the Problem Represented to Be?”
